# A study of the correlation between M2 macrophages and lymph node metastasis of colorectal carcinoma

**DOI:** 10.1186/s12957-021-02195-5

**Published:** 2021-03-29

**Authors:** Yanping Wang, Jikun Wang, Chunyu Yang, Yue Wang, Jinhao Liu, Zuoxiu Shi, Yanlei Chen, Yang Feng, Xueqian Ma, Shifeng Qiao

**Affiliations:** 1 The Second Ward of Colorectal Surgery, The First Affiliated Hospital of Jinzhou Medical University, No. 2, The Fifth Section of Renmin Street, Guta, Jinzhou, 121000 Liaoning People’s Republic of China; 2 Department of Oncology, The First Affiliated Hospital of Jinzhou Medical University, Jinzhou, 121000 Liaoning People’s Republic of China; 3 Department of Pathology, The First Affiliated Hospital of Jinzhou Medical University, Jinzhou, 121000 Liaoning People’s Republic of China

**Keywords:** M2 macrophages, CD163, Colorectal carcinoma, Lymph node metastasis, Carcinoembryonic antigen

## Abstract

**Background:**

Lymph node metastasis is a major prognostic sign of colorectal carcinoma and an important indicator for individualized treatment. M2 macrophages play a key role in carcinogenesis and tumor development by enhancing invasiveness and promoting lymph node metastasis. The purpose of this study was to investigate the effect of CD163-positive M2 macrophages on lymph node metastasis in colorectal carcinoma.

**Methods:**

Postoperative lymph node tissues were obtained from 120 patients with colorectal carcinoma who underwent radical surgery in the First Affiliated Hospital of Jinzhou Medical University between December 2019 and May 2020. We detected the expression of the CD163 protein in lymph nodes using immunohistochemistry. Furthermore, the relationships between M2 macrophages identified by expression of CD163 and lymph node metastasis were analyzed using the independent sample *t*-test and Chi-square test.

**Results:**

M2 macrophages were increased in metastatic lymph nodes and non-metastatic lymph nodes adjacent to the cancer. The M2 macrophage count was higher in patients with macro-metastases than in patients with micro-metastases.

**Conclusions:**

The presence of M2 macrophages represents an important indicator for lymph node metastasis in colorectal carcinoma and may be a potential marker for its prediction. Thus, M2 macrophage localization might offer a new target for the comprehensive treatment of colorectal carcinoma.

## Introduction

Colorectal carcinoma (CRC) is one of the most common malignant tumors worldwide, ranking third globally, with an annual incidence of approximately 1.2 million people. CRC results in more than 600,000 deaths annually, with an increasing mortality rate [1, 2]. The presence or lack of local lymph node metastasis (LNM) provides critical information on the tumor stage, clinical treatment, and patient prognosis. However, the survival rate for colorectal carcinoma patients with LNM is significantly worse than patients without LNM [3]. The mechanisms associated with the origin of LNM remain to be fully elucidated because LNM is a complex process. LNM involves numerous immune cells and changes in the expression of many different proteins that enable tumor cells to migrate from the primary lesion site, then travel to, adhere, and implant in the new environment. Compared to normal tissue, lymphatic drainage is increased in tumors. Regional lymph node immune tolerance is a necessary condition for the formation of LNM [4]. The tumor microenvironment (TME) provides a favorable location for the induction of immune tolerance. The TME is a highly complex network, which, in addition to tumor cells, includes a large number of immune cells, including macrophages, regulatory T (Treg) cells, natural killer cells, dendritic cells, T and B lymphocytes, and non-immune cells, such as endothelial cells, cancer-associated fibroblasts, and stromal cells. The relative proportion of cells in the TME is an essential factor influencing tumor cell invasion and metastasis [5]. Studies have shown that among these individual components, M2 macrophages play an essential role in promoting tumor growth, angiogenesis, lymphangiogenesis, immune tolerance, and anti-tumor immunity, and CD163 is a specific marker for these cells [6, 7].

Many studies have reported that the presence of large numbers of M2 macrophages in malignant tumor tissues such as gastric cancer, colorectal carcinoma, breast cancer, and cervical cancer is significantly correlated with decreased survival rates [8–11]. However, few studies have investigated the relationship between M2 macrophages and LNM. In the present study, the expression of CD163 in lymph node tissue was analyzed to explore the role of M2 macrophages in LNM in colorectal carcinoma, provide more accurate prognostic information, help identify new molecular therapeutic targets, and better understand the molecular mechanism of colorectal carcinoma progression.

## Materials and methods

### Patients and specimens

We collected clinical data and postoperative lymph node specimens from 120 patients with colorectal carcinoma treated at the First Affiliated Hospital of Jinzhou Medical University between December 2019 and May 2020. The clinical characteristics of all patients are shown in Table 1. Inclusion criteria were as follows. (1) The patient received a diagnosis of primary colorectal carcinoma. (2) The diagnosis occurred at our hospital, and the patient received surgical treatment for the first time. (3) The patient agreed to participate in this study. The exclusion criteria were as follows. (1) The patient had received chemotherapy or radiotherapy after the diagnosis. (2) The patient received targeted immune therapy after the diagnosis. (3) The patient exhibited two or more intestinal malignant tumors or other systemic malignant tumors. The lymph nodes obtained from the patients were divided into five groups as follows. Group A included 69 cases in which one normal lymph node was randomly selected from stage I and stage II patients. Group B included 51 cases in which one pathologically positive lymph node was randomly selected from stages III and IV patients. Group C included 51 cases in which one pathologically negative node was randomly selected from stages III and IV patients. Subsequently, all lymph nodes in group B were divided into two groups according to the size of the tumor in the lymph node. Thus, group D (*n* = 32) included lymph nodes that exhibited macro-metastasis (cell clusters > 2 mm) and group E (*n* = 19) included lymph nodes that exhibited micro-metastasis (cell clusters < 2 mm) [12]. One hundred and seventy-one lymph nodes were examined in this study. Clinical TNM staging was utilized according to the American Joint Committee on Cancer (AJCC) staging standard (8th edition). This study was approved by the Medical Ethics Committee of the First Affiliated Hospital of Jinzhou Medical University, and all patients signed informed consent forms.

**Table 1:** Patients’ characteristics

	Group A (*n*=69) (%)	Group B or C (*n*=51) (%)	Group D (*n*=32) (%)	Group E (*n*=19) (%)
Age				
Median (range)	67 (49~86)	63 (38~82)	62 (38~82)	64 (50~81)
Sex				
Male	40 (58)	35 (69)	22 (69)	13 (68)
Female	29 (42)	16 (31)	10 (31)	6 (32)
Anatomic tumor region				
Cecum	1 (1)	3 (6)	2 (6)	1 (5)
Ascending colon	9 (13)	11 (22)	6 (19)	5 (26)
Transverse colon	9 (13)	6 (12)	3 (9)	3 (16)
Descending colon	6 (9)	6 (12)	4 (13)	2 (11)
Sigmoid	14 (20)	9 (18)	6 (19)	3 (16)
Rectum	30 (44)	16 (30)	11 (34)	5 (26)
T stage (AJCC 8th)				
T1	1 (1)			
T2	18 (27)	3 (6)		3 (16)
T3	36 (52)	36 (71)	21 (66)	15 (79)
T4	14 (20)	12 (23)	11 (34)	1 (5)
N stage (AJCC 8th)				
N0	69 (100)			
N1		36 (71)	19 (59)	17 (89)
N2		15 (29)	13 (41)	2 (11)
Overall stage (AJCC 8th)				
I	19 (28)			
IIA	36 (52)			
IIB	10 (14)			
IIC	4 (6)			
IIIA		3 (6)		3 (16)
IIIB		32 (62)	19 (59)	13 (68)
IIIC		9 (18)	7 (22)	2 (11)
IV		7 (14)	6 (19)	1 (5)

### Immunohistochemical staining

All specimens were fixed in formalin, embedded in paraffin, and cut into 4-μm sections. The sections were placed on glass microscope slides, deparaffinized with xylene, and dehydrated in a graded ethanol series. Subsequently, antigen retrieval was performed using ethylenediaminetetraacetic acid buffer (pH 9.0) at a sub-boiling temperature for 20 min. The tissue sections were incubated with an endogenous peroxidase blocker at room temperature for 10 min, blocked with 3% goat serum (cat. No. KIT-9710; Maixin-Bio, Fuzhou, China) for 30 min, rinsed with phosphate-buffered saline (PBS), incubated with ready-to-use mouse anti-human CD163 monoclonal antibody (cat. No. MAB-0206; Maixin-Bio) at room temperature for 60 min, followed by sequential addition of biotin-labeled IgG polymer and streptavidin peroxidase (cat. No. KIT-9710; Maixin-Bio). Finally, the sections were developed using a diaminobenzidine (DAB) kit (cat. No. DAB-0031; Maixin-Bio) and counterstained with hematoxylin. The density of M2 macrophage infiltration in the lymph nodes was determined using bright-field microscopy (Olympus BX43; Japan). Two pathologists (Chun-yu Yang and Yue Wang) independently evaluated all the sections using a semiquantitative scoring system without knowing the patient’s clinicopathological data. The immunoreactive score was calculated as the staining intensity score × the score of the proportion of cell membrane that was stained. The staining intensity score was defined as 0, negative; 1, weak; 2, moderate; or 3, strong. The score for the proportion of cell membrane that was stained was defined as 0 (< 10%), 1 (11–25%), 2 (26–50%), 3 (51–75%), or 4 (76–100%). The total score ranged from 0 to 12. The final immunoreactive score was classified as positive (score > 4) or negative (score < 4) [13]. Subsequently, five high-power fields “hot spots” with M2 macrophage infiltration were randomly selected under low magnification (× 200), the number of M2 macrophages was counted (per mm^2^) under high magnification (× 400), and the mean number of M2 macrophages was calculated.

### Statistical analysis

The SPSS version 24.0 software program and GraphPad Prism 8 were used to analyze the data. Measurement data were expressed as means ± standard deviation. The independent sample *t*-test was used to compare differences in M2 macrophages in lymph node tissues with different clinicopathological parameters, as well as differences in M2 macrophages between groups. The Chi-square test was used to analyze the association between lymph node metastasis and clinicopathological parameters. Spearman correlation analysis was used to assess correlations between M2 macrophages and tumor markers. *P*<0.05 indicated statistically significant differences.

## Results

### Relationships between clinicopathological parameters and lymph node metastasis in colorectal carcinoma patients

As seen in Table 2, LNM in colorectal carcinoma patients was correlated with the degree of tumor differentiation, depth of invasion, and preoperative CEA, CA199, and CA724 levels (*P*<0.05), but not with gender, age, or tumor diameter (*P*>0.05).

**Table 2:** The correlation between the clinicopathological parameters and lymph node metastasis of colorectal carcinoma

Clinicopathological parameters	Number (*n*=120) (%)	Group A (*n*=69) (%)	Group B (*n*=51) (%)	χ^2^	*P* value
Gender				1.421	0.158
Male	75 (63)	40 (58)	35 (69)		
Female	45 (37)	29 (42)	16 (31)		
Age (years)				0.882	0.226
≥ 65	67 (56)	36 (52)	31 (61)		
< 65	53 (44)	33 (48)	20 (39)		
Tumor size (cm)				1.565	0.143
≥ 5	55 (46)	35 (51)	20 (39)		
< 5	65 (54)	34 (49)	31 (61)		
Preoperative CEA (ng/mL)				7.266	0.006
≥ 5	49 (41)	21 (30)	28 (55)		
< 5	71 (59)	48 (70)	23 (45)		
Preoperative CA199 (U/mL)				4.4004	0.038
≥37	27 (23)	11 (16)	16 (31)		
<37	93 (77)	58 (84)	35 (69)		
Preoperative CA724 (ng/mL)				28.434	0.000
≥6.9	35 (29)	7 (10)	28 (55)		
<6.9	85 (71)	62 (90)	23 (45)		
Differentiation degree				15.408	0.000
High/moderate	88 (73)	60 (87)	28 (55)		
Low	32 (27)	9 (13)	23 (45)		
T stage				4.310	0.031
T1 +T2	22 (18)	17 (25)	5 (10)		
T3 + T4	98 (82)	52 (75)	46 (90)		

### Expression of CD163 protein in lymph node tissues

**Fig. 1:**
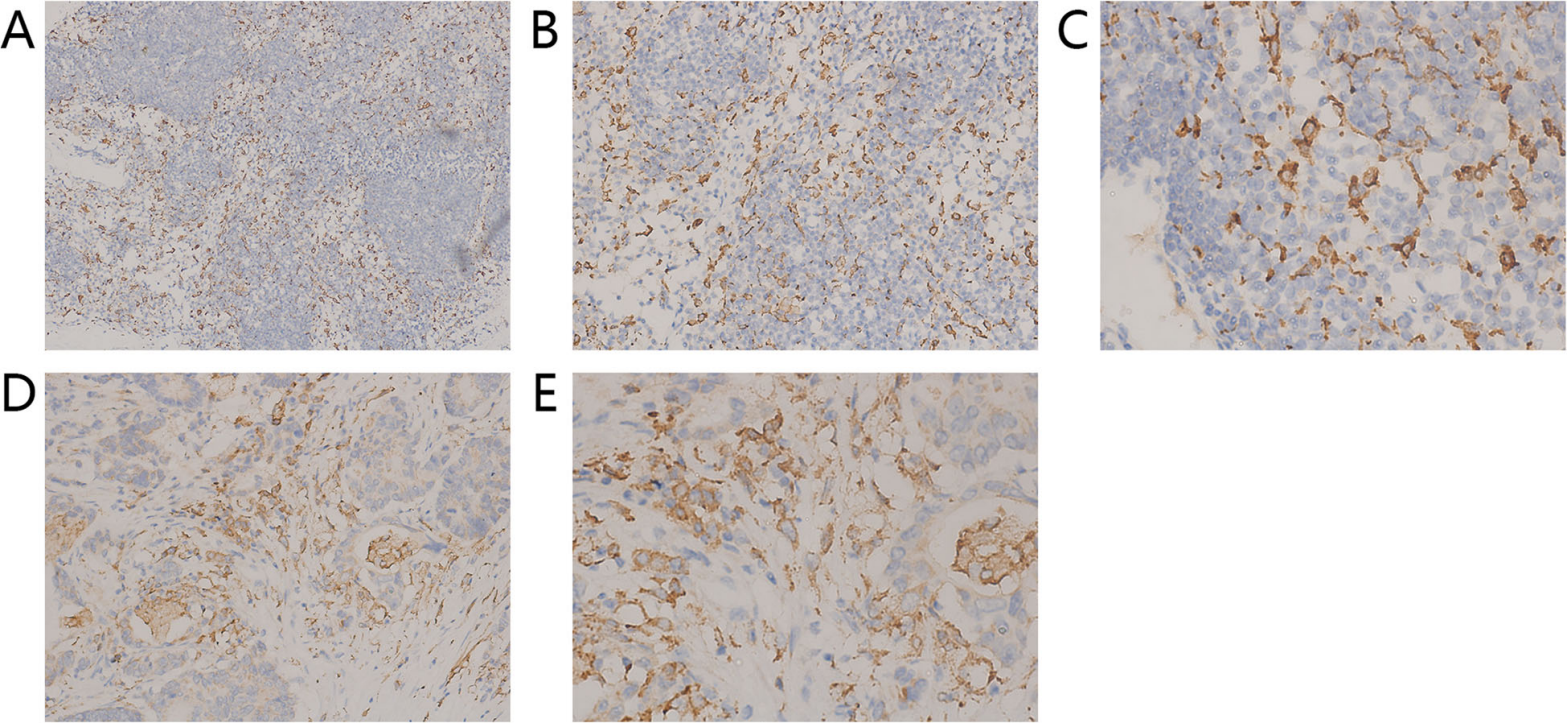
Expression of M2 macrophages in lymph nodes. Detection of CD163 in lymph nodes of colorectal carcinoma patients by immunohistochemistry. The membrane and cytoplasm of M2 macrophages are stained brown. Microscopic analysis of a typical example of CD163 expression in non-metastatic lymph nodes (**a**, **b**, **c** magnification × 100, × 200, × 400, respectively). **d**, **e** Location of CD163 in metastatic lymph node tissue at a magnification of × 200 and × 400 respectively. M2 macrophages are seen mainly infiltrating into the tumor stroma

Immunohistochemistry revealed that the expression of CD163 was characterized by the appearance of yellow or brown granules on the cell membrane of M2 macrophages, whereas cytoplasmic staining was slightly weaker. In group A (Fig. 1a, b) and group C (Fig. 1c), M2 macrophages primarily infiltrated the medullary sinus. In group B, M2 macrophages primarily infiltrated the peritumoral area, and relatively few M2 macrophages were seen in the intratumoral area (Fig. 1d, e).

### Mean numbers of M2 macrophages were different in different patient groups

**Fig. 2:**
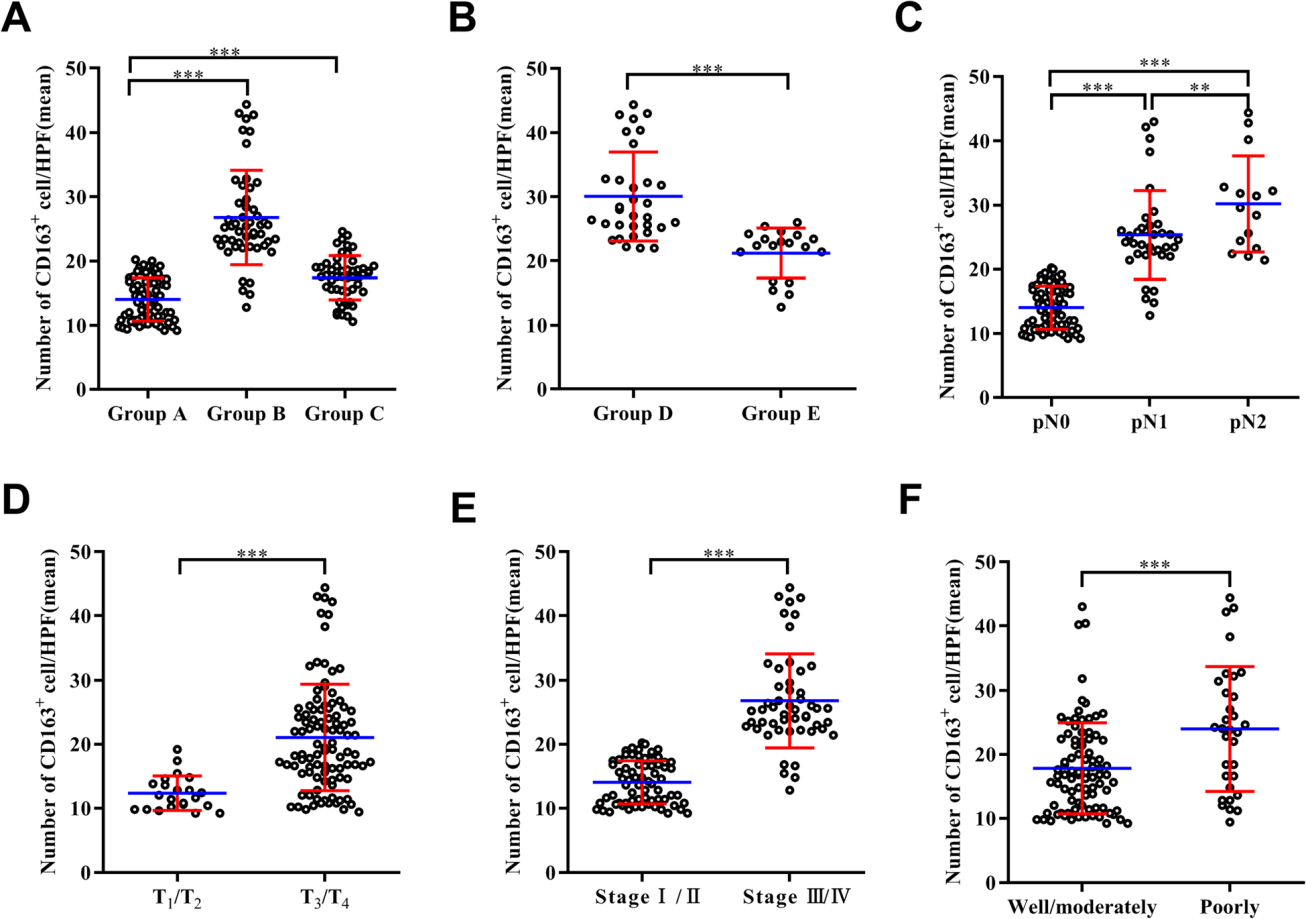
Comparison of the number of M2 macrophages in different groups of lymph nodes and the patients’ different clinicopathological features. Comparison of M2 macrophages in lymph nodes of different groups. **a** The mean number of M2 macrophages in metastatic lymph nodes (group B) is higher than that in normal lymph nodes (group A) (***P<0.0001), and the mean number of M2 macrophages in non-metastatic lymph nodes adjacent to cancer (group C) is higher than that in normal lymph nodes (group A) (***P<0.0001). **b** The mean number of M2 macrophages in macro-metastasis (group D) is higher than that in micro-metastasis (group E) (***P<0.0001). Comparison of M2 macrophages among different clinicopathological features. **c** The number of M2 macrophages is positively correlated with the degree of lymph node metastasis (***P<0.0001, **P=0.031). With the increase of pathological tumor (T) categories (**d**) and clinical TNM stage (**e**), the mean number of M2 macrophages also increased (***P<0.0001). **f** The greater the degree of tumor differentiation, the higher the mean number of M2 macrophages (***P<0.0001)

We observed that the mean number of M2 macrophages in group B (26.8±7.4) was significantly higher than group A (14.0±3.4) (Fig. 2a). However, it was not clear whether there was any difference in the number of M2 macrophages in lymph nodes between stages I and II patients and non-metastatic lymph nodes in stages III and IV patients. Therefore, we counted the M2 macrophages in groups A and C and found that the mean number of M2 macrophages in group C (17.4±3.4) was significantly higher than group A (14.0±3.4) (Fig. 2a). We also observed that the mean number of M2 macrophages in group D (30.0±7.0) was higher than group E (21.2±3.9) (Fig. 2b).

### Relationship between M2 macrophages in lymph node tissue and the clinicopathological features of colorectal carcinoma patients

To identify the characteristics of M2 macrophages infiltrating into lymph nodes, correlations between the mean number of M2 macrophages and patients’ clinicopathologic features were assessed. It was found that the increase in the mean number of M2 macrophages in lymph nodes paralleled the increase in pathological lymph node (N) categories (Fig. 2c). The mean number of M2 macrophages in the lymph nodes of patients with pathological tumor (T) categories was significantly different (Fig. 2d). In addition, the mean number of M2 macrophages in the lymph nodes of patients with stage III/IV colorectal carcinoma was significantly greater than stage I/II patients (Fig. 2e). Moreover, the mean number of M2 macrophages was greater in poorly differentiated metastatic lymph nodes than in moderately and well-differentiated lymph nodes (Fig. 2f).

### Correlations between the mean number of M2 macrophages in lymph node tissues and tumor markers

As shown in Table 3, Spearman analysis was used to identify correlations between the mean number of M2 macrophages and preoperative CEA, CA199, and CA724 levels. All levels were positively correlated with the mean number of M2 macrophages (*P*<0.05).

**Table 3:** Correlations of the mean number of M2 macrophages with CEA, CA19-9, and CA72-4 levels

Tumor markers	The mean number of M2 macrophages	
	Spearman correlation coefficient	*P* value
Preoperative CEA	0.337	0.001
Preoperative CA199	0.220	0.013
Preoperative CA724	0.171	0.041

## Discussion

The results of our studies demonstrated that increased numbers of M2 macrophages infiltrated into metastatic lymph nodes compared to non-metastatic lymph nodes. Also, more infiltration was seen in patients with macro-metastases than patients with micro-metastases. These observations indicate that M2 macrophages were closely associated with LNM in colorectal carcinoma. Also, M2 macrophages infiltrated into non-metastatic lymph nodes in patients who also exhibited metastatic lymph nodes, suggesting that the lymph node microenvironment had changed before metastasis. The results also suggested that M2 macrophages were involved in this process. Therefore, we speculated that M2 macrophages played a critical role in lymph node metastasis in colorectal carcinoma.

The occurrence and development of tumors are closely related to the TME. Generally, macrophages are the most abundant immune cell found in the TME, composing up to 50% of tumor stroma-infiltrating cells [14]. Because macrophages exhibit plasticity and functional diversity, they can be polarized into two types, M1 macrophages (classical activation) and M2 macrophages (alternative activation), depending on changes in the TME [15]. Agents such as lipopolysaccharide (LPS) and cytokines such as interferon-γ (IFN-γ) or granulocyte colony-stimulating factor (G-CSF) in the TME influence macrophage differentiation towards the M1 pathway. In contrast, macrophages exposed to anti-inflammatory cytokines such as IL-4, IL-10, IL-13, or TGF-β can be polarized into M2 macrophages. M1 macrophages produce a range of different pro-inflammatory cytokines, which kill pathogens and tumor cells and are useful for immune monitoring. M2 macrophages produce fewer pro-inflammatory cytokines but numerous anti-inflammatory cytokines, which causes immunosuppression and contributes to immune tolerance [16]. Previous studies on colorectal carcinoma indicated that a high M2:M1 ratio was closely related to enhanced tumor cell invasion [17]. Our study found that the number of M2 macrophages in lymph nodes was significantly correlated with the degree of tumor invasion, amount of differentiation, level of lymph node involvement, and the clinical TNM stage. These observations indicated that M2 macrophages were involved in forming an immunosuppressive environment in lymph nodes and are an important factor leading to lymph node metastasis, which was consistent with previous research results.

Tumor cell metastasis is a stage of deterioration in disease progression and is associated with a poor prognosis. Lymphatic metastasis is the most common form of tumor metastasis in various types of malignancies. We speculated that M2 macrophages might facilitate LNM based on several reasons. On the one hand, several studies [18–20] have shown that the number of lymphatic vessels in tumor tissues or metastatic lymph node tissues is significantly higher than normal tissues and is related to the presence of M2 macrophages. It has been confirmed that M2 macrophages produce VEGF-C, which induces lymphangiogenesis. Tacconi et al. [21] reported that VEGF-C binding to VEGFR3 on lymphatic vessels inhibited the expression of vascular endothelial cadherin (VE-Cad), resulting in damage to the endothelial barrier of lymphatic vessels around the tumor. This damage is conducive to the entry of tumor cells into lymphatic vessels. VEGF-C also promotes the proliferation and expansion of lymphatic vessels, which can increase the routes for tumor metastasis into lymph nodes [22]. Therefore, M2 macrophages might reshape the lymphatic network to provide favorable conditions for tumor cell metastasis.

On the other hand,there are a large number of cytokines in the TME, such as IL-4, EGF, IL-6, which can promote the polarization of M2 macrophages [23] in the TME. When IL-4 binds to IL-4R, it leads to phosphorylation of JAK-1 and JAK-3 and activates the downstream STAT6 signaling pathway [24]. Choi et al. [25] confirmed that STAT6 phosphorylation increased mRNA expression of M2 macrophage activation markers (FIZZ-1, ARG-1, and CD163). Conversely, inhibition of STAT6 signaling reduced the number of M2 macrophages. Yin et al. [26] found that the IL-6/JAK/STAT3 signaling pathway was inhibited during M1 macrophage polarization but activated during M2 macrophage polarization. Thus, a new mechanism of the IL-6/JAK/STAT3 signaling pathway regulating macrophage polarization was revealed. In addition, Lian et al. [27] noted that colon cancer cells secreted EGF and could bind to EGFR on monocytes, which activated the smad-PI3K-Akt-MTOR pathway and promoted monocyte differentiation into M2 macrophages. Therefore, when these factors described above are present in normal lymph nodes, they can promote macrophage polarization into M2 macrophages. M2 macrophages recruit Tregs into the TME by releasing chemokines (such as CCL 22 and CCL 24) [28]. Also, high expression of arginase-1,2 (ARG1,2) and indoleamine-2,3-dioxygenase 1 (IDO1) on the surface of M2 macrophages can greatly deplete arginine and tryptophan from the TME. Arginine and tryptophan are indispensable for the metabolism of immune cells, and their depletion leads to T cell and NK cell dysfunction [29–31]. Therefore, M2 macrophages enhance immunosuppression in lymph nodes to create favorable conditions for tumor cell metastasis.

Our study found that the mean number of M2 macrophages gradually increased from 9.2 in normal lymph node tissue to 44.4 in metastatic lymph node tissue. We also observed that M2 macrophages primarily infiltrated into the peritumoral region, but relatively few were present in the intratumoral area. Therefore, we speculated that numerous M2 macrophages infiltrated around tumor cells, leading to the formation of an immunosuppressive TME and remodeling of the lymphatic network, which enhanced invasion of tumor cells and promoted metastasis. We also found that preoperative CEA, CA724, and CA199 levels were closely related to lymph node metastasis, and CEA was closely related to the number of M2 macrophages present. Therefore, we speculated that tumor cells might promote the differentiation of M2 macrophages through CEA secretion, which promoted lymph node metastasis.

In this study, we comprehensively analyzed the presence of M2 macrophages in lymph node tissues of patients with different stages of colorectal carcinoma and assessed the possible relationships. We found that M2 macrophages were more numerous in metastatic lymph nodes and non-metastatic lymph nodes in patients with lymph node metastasis. Therefore, we speculated that M2 macrophages were important components in the process of lymph node metastasis in patients with colorectal carcinoma. Although the specific molecular mechanism whereby M2 macrophages contributed to metastasis in colorectal carcinoma was not clear, the results of this study provide a foundation for future research. M2 macrophages exhibit multiple surface markers, including CD68, CD163, and CD206. However, CD68 is a pan-macrophage marker and cannot distinguish between M1 and M2 macrophages. Also, CD206 is expressed not only on macrophages and dendritic cells but also on lymphatic vessels, hepatic cells, and splenic endothelial cells. CD163 is a type I transmembrane protein with a molecular weight of 130kD. It consists of nine cysteine receptor domains and is a type B scavenger receptor member. It is mainly used to identify M2 macrophages, and its role in the polarization of M2 macrophages is well understood [17, 32, 33]. Therefore, CD163 was selected as the marker for M2 macrophages in our study.

In conclusion, M2 macrophages might alter the tumor microenvironment and promote lymph node metastasis in colorectal carcinoma. Our findings provide a reference for understanding lymph node metastasis of colorectal carcinoma and future development of treatment targets.

## Data Availability

The analyzed data sets generated during the study are available from the corresponding author on reasonable request. Inquiries for data access may be sent to the following e-mail address: shifengqiao2020@163.com.
